# Quantifying root colonization by a symbiotic fungus using automated image segmentation and machine learning approaches

**DOI:** 10.1038/s41598-023-39217-z

**Published:** 2023-09-08

**Authors:** Ivan Sciascia, Andrea Crosino, Andrea Genre

**Affiliations:** Department of Life Sciences and Systems Biology, Università di Torino, Turin, Italy

**Keywords:** Plant sciences, Image processing

## Abstract

Arbuscular mycorrhizas (AM) are one of the most widespread symbiosis on earth. This plant-fungus interaction involves around 72% of plant species, including most crops. AM symbiosis improves plant nutrition and tolerance to biotic and abiotic stresses. The fungus, in turn, receives carbon compounds derived from the plant photosynthetic process, such as sugars and lipids. Most studies investigating AM and their applications in agriculture requires a precise quantification of the intensity of plant colonization. At present, the majority of researchers in the field base AM quantification analyses on manual visual methods, prone to operator errors and limited reproducibility. Here we propose a novel semi-automated approach to quantify AM fungal root colonization based on digital image analysis comparing three methods: (i) manual quantification (ii) image thresholding, (iii) machine learning. We recognize machine learning as a very promising tool for accelerating, simplifying and standardizing critical steps in analysing AM quantification, answering to an urgent need by the scientific community studying this symbiosis.

## Introduction

Arbuscular mycorrhizas (AM) are widespread plant endosymbioses that develop between Glomeromycotina fungi and the roots of the majority of plant species, including most crops. The symbiosis benefits extend to both partners by improving plant mineral absorption, tolerance to biotic and abiotic stresses and fitness, while rewarding the fungal symbionts with carbohydrates compounds derived from photosynthesis process, such as sugars and lipids^1^. The nutrient exchange represents the functional core of the symbiosis and occurs at the contact surfaces of highly branched fungal structures—called arbuscules—that are hosted within the living cortical cells of the plant roots^2^. The central ecological role of AM in the functioning of low-input ecosystems and the ability of most crop plants to develop this symbiosis has focused a growing number of investigations on the use of AM in sustainable agricultural practices. A critical step in all studies on AM is represented by the precise quantification of root colonization by AM fungi, with particular attention to arbuscule abundance^3^. Nevertheless, molecular analyses, based on the quantification of fungal sequences in total root DNA or arbuscule-specific markers in total root RNA extracts, are still outnumbered by direct microscopic quantification of the intraradical fungal structures, after histochemical staining^4, 5^. In one of the most commonly used methods, root samples are stained with lactic blue or alternative dyes to label intraradical fungal structures; roots are then cut into 1 cm-long segments, mounted on microscope slides and carefully observed under an optical microscope to classify each segment, based on visual criteria such as the extension of intraradical hyphae and the abundance of arbuscules in the colonized areas^6^. Such methods are extremely time consuming, based on the ability of trained operators and subject to errors.

An emerging alternative, improving speed, repeatability, and reliability of root colonization measurements, is offered by automated pixel-based classification of digital images from optical microscopy. This can be achieved either with a traditional method, known as thresholding, which classifies pixels according to their grayscale intensity, or using a more complex analysis, based on machine learning, where each pixel is described by a set of parameters extracted from the environment of neighbouring pixels.

- Our study addressed this topic by comparing the commonly used visual method developed by Trouvelot et al.^6^ with semi-automated algorithms to generate quantitative indexes of root colonization.
- We developed an image thresholding approach using ImageJ.
- We applied an innovative approach based on machine learning^7^, taking advantage of the commercial software Zeiss Intellesis^8^.
- Our analyses identify machine learning as the most promising alternative to visual quantification of AM root colonization.

## Materials and methods

**Figure 1:**
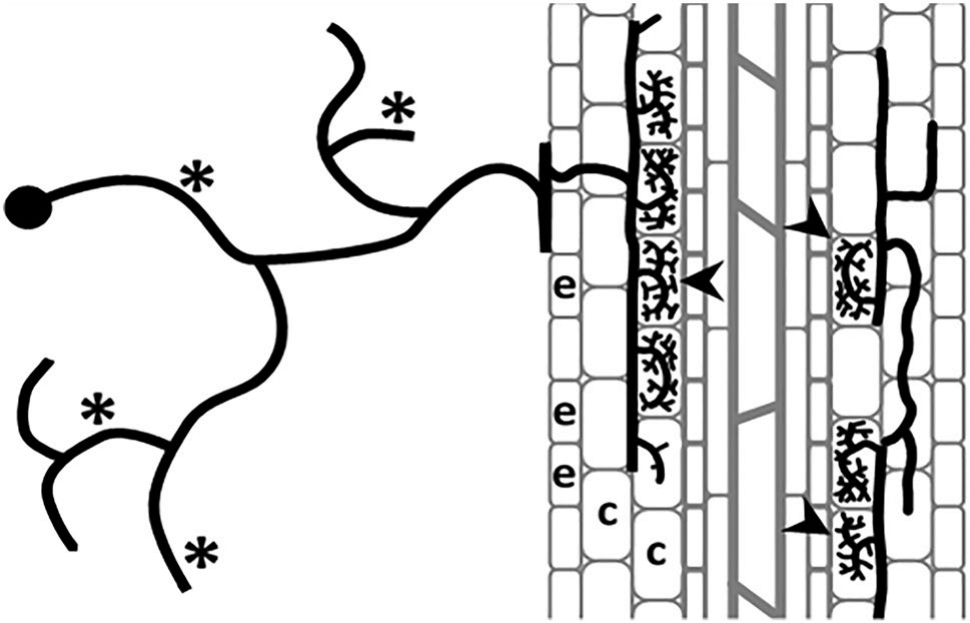
Schematic representation of a host root (grey) colonized by an arbuscular mycorrhizal fungus (black). The extraradical mycelium (*) explores the soil surrounding the root, while intraradical structures produced from the hyphopodium (h) penetrate root epidermal cells (e), colonizing single cortical cells (c), where they eventually develop into branched arbuscules (arrowheads), the sites of nutrient exchanges between symbionts.

To quantify the extent of AM fungal root colonization, we acquired a dataset of 180 root images of *Solanum pennellii* colonized with the AM fungus *Funelliformis mosseae* and not colonized, stained with 0.1% methylene blue in lactic acid. Quantitative AM fungal colonization first was measured based on the frequency and abundance of fungal structures following Trouvelot et al.^6^ as described in Volpe et al.^9^ considering it as the (i) visual method. A schematic representation of colonization process and structures is described in Fig. 1. The images were acquired with CI-L fitted with a 4x/0.10 WD30 objective or Leica DMA500 fitted with a PLAN4x/0.10 objective. The fungal structures were stained blue while the plant tissues and cells remained transparent or light blue.

### Visual method

Fungal presence could thus be detected based on contrast in staining intensity. The dataset of 180 images was visually scored for six root colonisation intensity classes, ranging from not mycorrhizal to > 90% mycorrhizal roots, using the procedure of Trouvelot et al.^6^. Each fragment is then assigned to a mycorrhization class, according to the following criteria:

- Class 1 without infection.
- Class 2 few traces.
- Class 3 less than 10%.
- Class 4 from 11 to 50%.
- Class 5 from 51 to 90%.
- Class 6 more than 90%.

In particular, the method of Trouvelot et al.^6^ and Brundrett et al.^10^ are based on non-vital staining of fungal cell wall in mycorrhizal roots, followed by the visual ranking of a representative sample of root segments and a statistical analysis of the results, to extrapolate whole root system estimates.

### Digital image thresholding

A multicolour digital image can be represented by three components^11^: red, green and blue (RGB) and the shades of each colour can be represented by ${2}^{8} \to \left[\mathrm{0,255}\right]$ bits (1byte). A digital image is therefore a function of the type:

$$f:D \to [\mathrm{0,255}]$$

where D is a spatial domain composed of coordinates $\left(x,y\right)$ in a sampling grid, each element of which is called a pixel.

**Figure 2:**
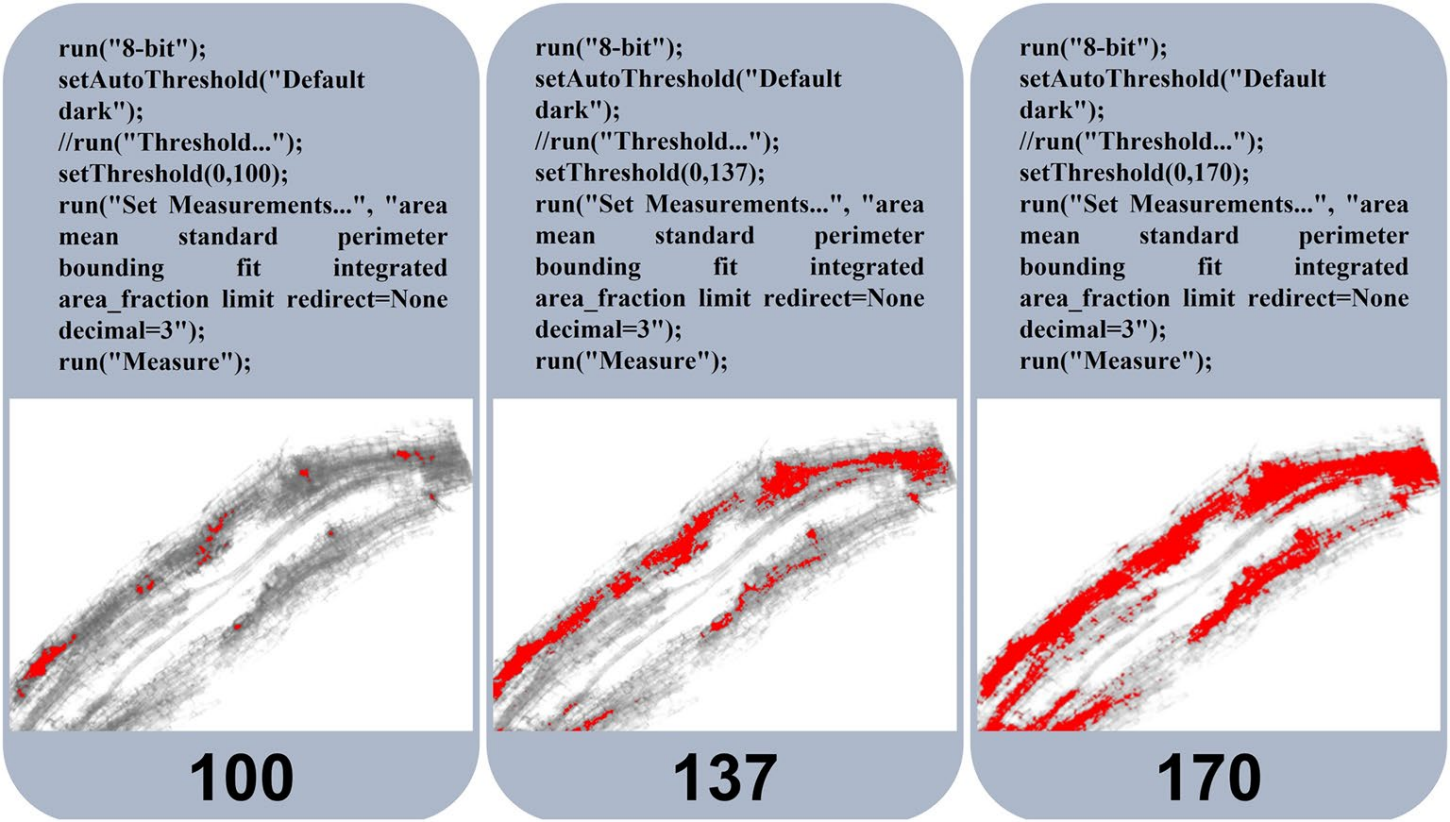
Pixel brightness-based thresholding of the same image from a mycorrhizal root segment. Pixels are selected (in red) based on arbitrary thresholds set at 100, 137, 170 in a range from 0 (black) to 255 (white) using Fiji/ImageJ. Above images, the corresponding Fiji/ImageJ macros are shown.

For this approach we used the Fiji/ImageJ software^12^. After converting each RGB channel into a grayscale image, a threshold can be set to select all pixels whose brightness is below the given value. The software will then measure the area of the selected pixels (Fig. 2).

After applying the segmentation macro to all our images, a set of quantitative values was obtained, corresponding to the supposed colonised area (darkest pixels) and the total root section area (as isolated from the image background). For statistical analysis, the thresholding (t) index was considered, which referred to root colonisation intensity based on contrast thresholding and expressed as the ratio between the mycorrhized area and the total area. Statistical analyses were carried out with the SPSS software (IBM Statistics for Windows version 26.0).

$$t=\frac{mycorrhized \, area}{total \, area}*100$$

### Machine learning

As our last approach, we tested an image analysis procedure, based on machine learning, using Zeiss Zen Intellesis application (Carl Zeiss Microscopy GmbH Jena, Germany)^13^. Machine learning algorithms are raising increasing interest in computer vision based applications^14, 15^. Such approaches are a branch of artificial intelligence, which allow to solve tasks using algorithms that are capable of learning from experience (training), without being explicitly programmed for a specific task.

As specified in the introduction section, the method we used in this analysis was based on the characteristics of neighbouring pixels that were subsequently classified based on description vectors. The Zeiss Intellesis software proposes up to 7 different machine learning techniques. For this analysis, the software developers suggested to apply the basic feature 25 algorithm, as the most suitable for this type of explorative study. In fact, this algorithm applies a series of feature descriptors (Gaussian Filter using 5 parameters, Sobel of Gaussians for 5 parameters, Gabor Filter for 12 parameters and Hessian Filter for 3 parameters) to create a final description vector composed of 25 parameters^16^. The algorithm training phase was performed from the same operator who manually classified the image dataset. During this phase, individual fungal structures such as intracellular hyphae and arbuscules were manually selected to generate a model that the software then applied to the whole image dataset.

Also in this case, we developed a root colonization intensity index (machine learning index, ml-index) as the ratio between the colonized area and the sum between the colonized area and the not colonized area:

$$ml=\frac{colonized \, area}{colonized \, area+non \, colonized \, area}*100$$

## Results

### Digital image thresholding

As described in the “Materials and Methods” section, a dataset composed of 180 colonized and uncolonized root images was processed, setting the intensity threshold to 100 in our Fiji/ImageJ macro previously mentioned. Once the t index was extrapolated, different statistical analyses were performed, using the SPSS software.

Table 1 shows the descriptive parameters of the t index (mean, standard deviation and range) for the comparison between the categories identified by the thresholding method and those obtained after visual classification. The subsequent ANOVA variance analysis revealed a highly significant statistical difference in the distribution of the t index between the six classes (Table 2). Furthermore, the ANOVA pairwise correlation analysis (Table 3) confirmed that the thresholding method allowed a significant level of discrimination for 12 out of 15 pairwise comparisons.

**Table 1:** Variability of the *t*-index comparing thresholding analysis and visual classification.

Visual classification	Number of images	Colonization (%)	Mean (t)	St. dev (t)	Range (t)
1	30	0	1.01	1.19	5.58
2	30	< 5	5.87	4.40	16.44
3	30	5–10	14.39	10.19	38.98
4	30	11–50	15.17	8.36	28.64
5	30	51–90	29.75	13.22	51.33
6	30	> 90	29.97	11.68	45.35
Total	180		16.03	14.20	60.32

**Table 2:** ANOVA variance for the six groups of digital images for the t index.

Variance	Sum of squares	df	Mean square	F	Probability
Between groups	21,439.36	(5)	4287.87	(50.82)	(< 0.01)
Within groups	14,680.18	(174)	84.36		
Total	36,119.54	(179)

**Table 3:** ANOVA pairwise correlation analyses of the root colonisation intensities as inferred by the t-index in the visual categories.

Visual categories	1	2	3	4	5	6
1		4.86	13.38*	14.16*	28.74*	28.95*
2			8.51*	9.29*	23.87*	24.09*
3				0.77	15.35*	15.57*
4					14.57*	14.79*
5						0.21
6						

Statistics with pairwise post hoc multiple comparisons (Bonferroni method). *Mean difference is significant at 0.05 level.

**Figure 3:**
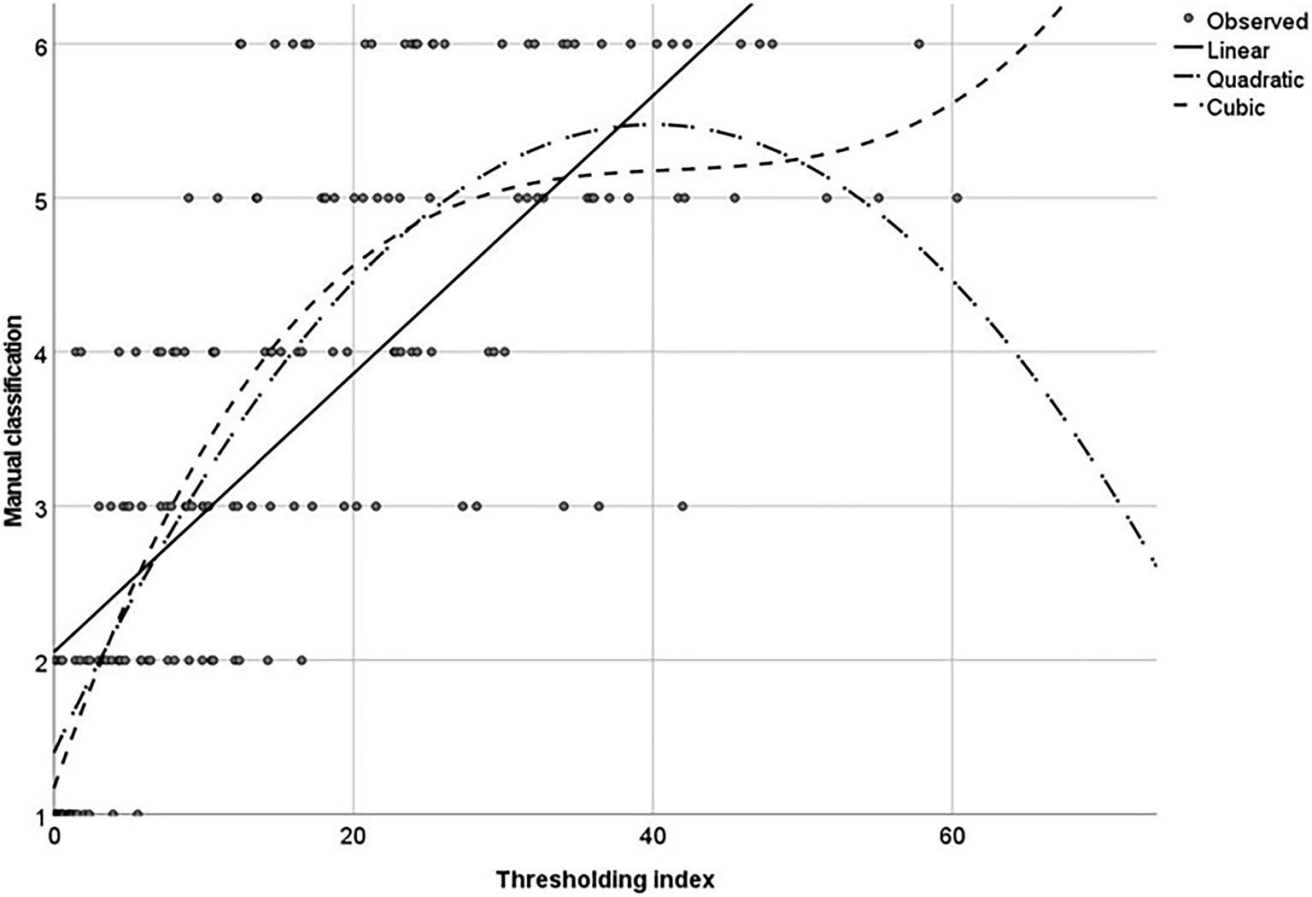
Root colonisation intensities based on t index distribution (x axis) and manual image scoring (y axis). Cubic fit R^2^ = 0.687.

We then performed a regression analysis to study the fitness of theoretical functions to our experimental data (Fig. 3). This analysis showed that the cubic model:

$${y}_{t \, index}=a+{b}_{1}x+{b}_{2}{x}^{2}+{b}_{3}{x}^{3}$$

better fits with the experimental distribution (R^2^ = 0.687).

### Machine learning

In analogy with the thresholding method, we calculated the descriptive parameters (Table4), ANOVA variance analysis (Table 5) and ANOVA pairwise comparisons (Table 6) for the ml-index.

**Table 4:** Variability of the *ml*-index based on manually processed images.

Visual classification	Number of images	Colonization (%)	Mean (mL)	St. dev (mL)	Range (mL)
1	30	0	6.42	6.20	18.60
2	30	< 5	7.88	8.64	41.81
3	30	5–10	17.30	11.75	40.66
4	30	11–50	26.79	10.59	49.60
5	30	51–90	44.15	11.37	37.43
6	30	> 90	48.43	8.84	33.87
Total	180		25.16	19.06	65.74

**Table 5:** ANOVA variance for the six groups of digital images for the *ml*-index.

Variance	Sum of squares	df	Mean square	F	Probability
Between groups	48,474.66	(5)	9694.93	(101.84)	(< 0.01)
Within groups	16,563.85	(174)	95.19		
Total	65,038.52	(179)

**Table 6:** ANOVA pairwise correlation analyses of the root colonisation intensities as inferred by the ml-index in the visual categories.

Visual categories	1	2	3	4	5	6
1		1.46	10.87*	20.36*	37.72*	42.00*
2			9.41*	18.90*	36.26*	40.54*
3				9.49*	26.84*	31.12*
4					17.35*	21.63*
5						4.27
6						

Statistics with pairwise post hoc multiple comparisons (Bonferroni method). *Mean difference is significant at 0.05 level.

Also in this case, the ANOVA variance analysis showed a statistically significant difference in the distribution of the ml index between the six classes (Table 5), which was confirmed by the ANOVA pairwise analysis, highlighting 13 significant comparisons out of 15 (Table 6).

**Figure 4:**
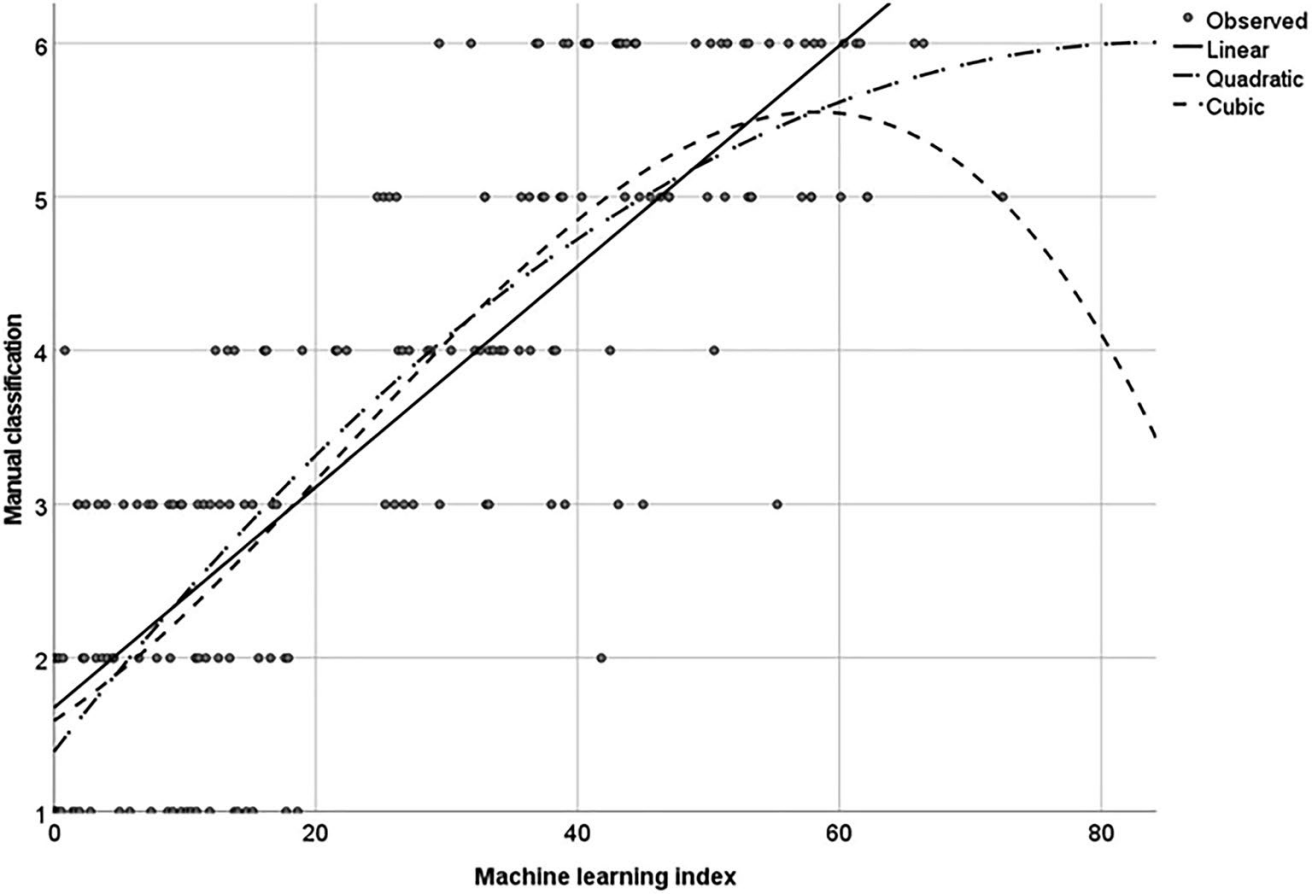
Root colonisation intensities as determined by simple image machine learning and as classified by manual image scoring. Cubic fit R^2^ = 0.728.

Furthermore, a prediction model for the level of mycorrhization was also built for the machine learning method, revealing that the cubic model:

$${y}_{ml \, index }=a+{b}_{1}x+{b}_{2}{x}^{2}+{b}_{3}{x}^{3}$$

best fits the experimental data (Fig. 4) with R^2^ = 0.728.

In conclusion, a comparison between the two semi-automated methods (Table 7) indicated the machine learning method based on the Zeiss Zen Intellesis application as the most efficient in discriminating between image classes, with a very high correlation (Pearson correlation coefficient 0.824) with manual analysis.

**Table 7:** Model performances comparisons.

Performance index	Thresholding	Machine learning
R^2^ linear	0.56	0.71
R^2^ quadratic	0.673	0.72
R^2^ cubic	0.687	0.728
Significant*	12/15	13/15

*Significant pairwise post hoc multiple comparisons on total comparisons, Bonferroni method. Mean difference is significant at 0.05 level.

## Discussion

The degree of root colonization is a fundamental parameter in most studies on AM. Assessing an extent of AM fungal root system provides a direct indication of symbiosis development and functioning. Indeed, quantitative estimates of AM colonization are a pre-requisite for studies reporting promotion of plant nutrition and growth by the AM symbiosis^17^. Two main approaches are currently used to quantify root colonization: molecular- and microscopy-based quantification^18^. Overall, the molecular approach is relatively fast and sensitive to quantify the fungal abundance, but cannot discriminate among fungal structures (e.g. arbuscules and hyphae), which limits its suitability when studying symbiosis functioning, unless used in combination with functional markers, such as plant P or Zn transporters that are only expressed in arbusculated cells^3^.

By contrast, microscopic methods, albeit time consuming, provide more direct information on AM development and are therefore of common use.

Even if it is based on objective traits, microscopic quantification is subject to observer bias and performs best when the same person analyses all samples^19^. The present study thus evaluated the reliability of two semi-automated image analysis methods in comparison to manual scoring^6, 20^ demonstrating that image analysis is suitable for ranking samples according to root colonization intensity, in analogy with recent studies^21, 22^.

The thresholding method uses the gradient of pixel brightness (inversely related to cotton blue staining) as an indicator of fungal presence. Quantification of root colonization by thresholding resolved the six root colonization intensity classes, as for the visual scoring, and could therefore be considered reliable for rapidly screening root samples. A few critical aspects should anyway be considered. One major limitation of the thresholding method was the variability of brightness range between images: different dyes, optical setups, root translucence and the presence of additional microorganisms (such as bacteria, algae, endophytic fungi, invertebrates) especially from field samples, often cannot be discriminated from fungal structures simply based on pixel brightness. In addition, the method is strongly affected by image background noise and magnification. Lastly, the segmentation process can only be set *ex ante*, by changing the macro settings without subsequent adjustments by the user.

The machine learning-based procedure of the Zeiss Zen Intellesis resulted to be the most efficient. It allowed the discrimination among the different fungal structures, such as hyphae, arbuscules and vesicles, based on the manual training phase, and generated a model that the software then applied to all analysed samples. This approach also resolved the six classes of intensity and achieved the best correlation with manual colonization scoring. Importantly, training phase was relatively short (it required 50 min overall) and resulted to be effective, even when using a limited number of images (10 images). Lastly, the machine-learning performed a reliable discrimination between intra- and extraradical hyphae, as well as intraradical hyphae and arbuscules, a major advantage compared to the pixel brightness thresholding method.

## Perspectives and conclusions

A critical factor for accurate machine learning-based root colonization level assessment is that the software should be trained by an expert operator. Nevertheless, the Zeiss Intellesis software allows the storage of all images used for the training phase in a reference folder. This set of images can therefore be shared with other researchers and integrated with additional reference images. This opens a new perspective for data reproducibility: research groups can share their expertise with the scientific community by simply sharing the images in a public online repository. Furthermore, the training file can also become a shared resource for reference and make the quantification of AM colonization more uniform and repeatable between different laboratories. The current model of machine learning could be implemented to reach beyond the simple assessment of mycorrhization intensity classes, but also reliably quantify the presence of arbuscules, vesicles, hyphal coils and so forth. Furthermore, the versatility of this approach opens new perspectives and possibilities regarding its application to other plant interactions such as those with endophytic fungi^23^. At present, the commercial nature of the software hampers the modification of the image analysis algorithm, unlike open-source software such as the one used by Evangelisti et al.^21^. Nevertheless, a commercial software has the advantage of regular and coordinated upgrades by the producer. Recently, two other AM quantifying machine learning based methods were developed, based on (and aimed to replace) a different manual quantification method, the so-called grid line intersection technique^21, 22^. Indeed, such methods were described to reliably discriminate among fungal structures (arbuscules, hyphae and vesicles). It would be extremely interesting to investigate the possibility to merge the three algorithms in an attempt to develop a more powerful tool for image analysis that could make quantification of root colonization by AM fungi more reproducible and with more efficient structure discrimination.

## Data Availability

The analysed datasets are available from Figshare: Segmentation using thresholding and machine learning of mycorrhizal roots. https://doi.org/10.6084/m9.figshare.14679729. Segmentation using thresholding and machine learning of non mycorrhizal roots. https://doi.org/10.6084/m9.figshare.14679684.
